# Discovering a rotational barrier within a charge-transfer state of a photoexcited chromophore in solution

**DOI:** 10.1063/1.5143441

**Published:** 2020-03-04

**Authors:** Taylor D. Krueger, Sean A. Boulanger, Liangdong Zhu, Longteng Tang, Chong Fang

**Affiliations:** Department of Chemistry, Oregon State University, 153 Gilbert Hall, Corvallis, Oregon 97331, USA

## Abstract

Methylation occurs in a myriad of systems with protective and regulatory functions. 8-methoxypyrene-1,3,6-trisulfonate (MPTS), a methoxy derivative of a photoacid, serves as a model system to study effects of methylation on the excited state potential energy landscape. A suite of spectroscopic techniques including transient absorption, wavelength-tunable femtosecond stimulated Raman spectroscopy (FSRS), and fluorescence quantum yield measurements via steady-state electronic spectroscopy reveal the energy dissipation pathways of MPTS following photoexcitation. Various solvents enable a systematic characterization of the H-bonding interaction, viscosity, and dynamic solvation that influence the ensuing relaxation pathways. The formation of a charge-transfer state out of the Franck–Condon region occurs on the femtosecond-to-picosecond solvation timescale before encountering a rotational barrier. The rotational relaxation correlates with the H-bond donating strength of solvent, while the rotational time constant lengthens as solvent viscosity increases. Time-resolved excited-state FSRS, aided by quantum calculations, provides crucial structural dynamics knowledge and reveals the sulfonate groups playing a dominant role during solvation. Several prominent vibrational motions of the pyrene ring backbone help maneuver the population toward the more fluorescent state. These ultrafast correlated electronic and nuclear motions ultimately govern the fate of the photoexcited chromophore in solution. Overall, MPTS in water displays the highest probability to fluoresce, while the aprotic and more viscous dimethyl sulfoxide enhances the nonradiative pathways. These mechanistic insights may apply robustly to other photoexcited chromophores that do not undergo excited-state proton transfer or remain trapped in a broad electronic state and also provide design principles to control molecular optical responses with site-specific atomic substitution.

## I. INTRODUCTION

Methylation is a rather simple molecular substitution that has far-reaching implications in chemical and biological systems.^1,2^ In synthetic chemistry, the reduced reactivity of the methyl substituent can protect against undesired reactions.^3^ Methylation is also prevalent as a post-translational modification in natural systems from proteins to genomes. The methylation of DNA is essential to mammalian development through a myriad of complex regulatory roles, and the methylation cycle in the human body can be improved by specific nutrients such as B-complex vitamins.^4,5^ From a fundamental perspective with detailed molecular dynamics insights, the 8-methoxypyrene-1,3,6-trisulfonate (MPTS) serves as an excellent model system to study the effect of methylation on the energy dissipation pathways within the electronic excited state.

The parent molecule 8-hydroxypyrene-1,3,6-trisulfonic acid (HPTS or pyranine) is a well-studied photoacid whose p*K*_a_ drops from ∼7 to 0 from the electronic ground state (S_0_) to the first singlet excited state (S_1_).^6–13^ The significant drop in p*K*_a_ following photoexcitation is due to the phenolic hydroxy bond weakening upon electronic redistribution, which precedes a swift proton dissociation event. Such an excited state proton transfer (ESPT) reaction, with strong implications in photosynthesis and energy harvesting, is one of the most fundamental reactions that plays important roles in chemical, biological, and energy-related systems.^14–17^ Therefore, HPTS has been used as a model system to dissect the ESPT mechanisms in many spectroscopic studies. Notably, since the methylation of HPTS inhibits ESPT, the resultant MPTS allows for a comparative study of the photophysical properties of the pyrene derivatives to reveal the ultrafast elementary steps leading to proton transfer. As a common control sample with a similar structure and energetics to HPTS, the electronic and vibrational spectra of MPTS have been reported,^11,18–20^ yet the underlying molecular structural dynamics remain ambiguous because the primary focus was ESPT. On the other hand, MPTS offers several opportunities to elucidate the photophysics and photochemistry of the π-conjugated systems with site-specific substitutions. In this work, an integrated time-resolved electronic and vibrational spectroscopic platform was employed to expose the effects of single-site methylation of a photoacid on the excited-state potential energy surface (PES). In particular, femtosecond transient absorption (fs-TA) spectroscopy and femtosecond stimulated Raman spectroscopy (FSRS) can be used in tandem to provide new structural dynamics insights.

Previous reports revealed ultrafast dynamics of the photoacid (PA) form of HPTS in water with typical time constants of ∼300 fs, 2.5 ps, and 90 ps, assigned to an initial charge separation, contact ion-pair formation, and rotational diffusion-assisted further ion separation after ESPT, respectively.^8,11,21^ The presence and terminology of the contact ion-pair have been debated;^12,22^ it may be better categorized as an intermediate solute-proton-solvent complex displaying an electronic state with discernible spectral features vs PA^*^ (asterisk means the electronic excited state) and PB^*^ (deprotonated chromophore in S_1_).^16,23,24^ Moreover, the photoinduced charge separation and subsequent rotational diffusion of the chromophore are generally accepted steps for the excited state relaxation. Regarding MPTS, though the proton-relevant diffusional stage is absent without ESPT, a chromophore rotational component (∼130 ps) on a similar timescale to HPTS (∼90 ps) was observed for MPTS in water via fluorescence anisotropy measurements.^25^ Using the time-resolved fluorescence techniques to track the intrinsic anisotropy being depolarized by an exponential decay function, Huppert and coworkers recently studied the rotational relaxation time of HPTS and MPTS. They found a shorter than previously reported time constant (∼80 ps) in water for both and a much longer than expected time constant (190 ps) for HPTS in methanol.^12^

With no chemical reaction expected to occur for MPTS in solution, the pertinent model may be “what goes up must come down,” a simplified description of photoexcitation and subsequent relaxation. In particular, a rotational phase was observed for HTPS and MPTS, but how does this component fit into energy dissipation pathways? Is it a nonradiative transition to the ground state or an excited-state relaxation? We address these questions by studying MPTS via an integrated experimental toolset including the steady-state electronic spectroscopy, fs-TA,^26^ and tunable FSRS.^16,27,28^ Fluorescence quantum yield (FQY) measurements were taken in three solvents to correlate the ultrafast dynamics to the steady state, aided by quantum calculations.^29^ Notably, three excited-state processes govern the excited state dynamics: solvation, rotational relaxation, and fluorescence. All three processes are intimately affected by the polarity, hydrogen (H-)bonding ability, and viscosity of the solvents used: water (H_2_O), methanol (MeOH), and dimethyl sulfoxide (DMSO). This investigation of MPTS reveals a more nuanced PES than that initially predicted, while providing insights into various roles played by rotational motions of a chromophore in solution.

## II. RESULTS AND DISCUSSION

### A. Steady-state electronic spectroscopy of MPTS in solution

**FIG. 1.**
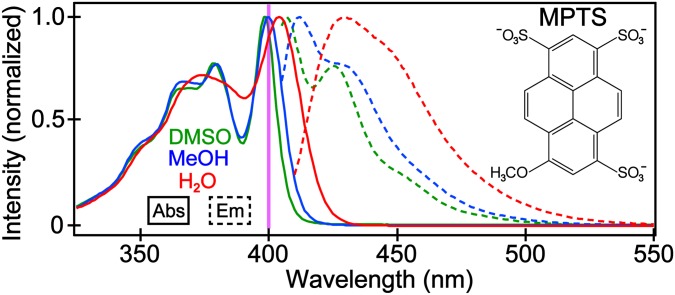
The normalized steady-state absorption (solid) and emission (dashed) spectra of MPTS in DMSO (green), MeOH (blue), and H_2_O (red). The vertical purple line indicates the excitation wavelength for fluorescence measurements, and the ensuing TA and FSRS experiments. The MPTS molecular structure is shown in the inset.

The absorption and fluorescence spectra of MPTS (Fig. 1) display similar profiles in three representative (e.g., polar, protic, and aprotic) solvents. The main electronic S_0_ → S_1_ transition at ∼400 nm is accompanied by several broad peaks to the blue side likely due to vibronic progression and an adjacent electronic transition band.^18,30^ Since MPTS only has H-bond accepting ability, the solvatochromism is primarily evidenced by the absorption peak redshift as the solvent H-bond donating strength increases.^31–33^ DMSO has the least H-bond donating ability while H_2_O has the strongest, resulting in the λ_max_ of 398 and 404 nm, respectively (Table I). Due to the photoinduced charge transfer (CT) from the methoxy site toward the ring system and sulfonate groups of MPTS,^11,18,19^ the enhanced H-bond accepting capabilities of the solute molecule in S_1_ vs in S_0_ should be concentrated on the three sulfonate groups (see Sec. II E below for further experimental corroboration). The correlation between the absorption wavelength and the overall H-bonding strength can be explained, in part, by the particle-in-a-box model: the better the H-bonding network, the larger the quantum box for electrons, hence shifting the absorption spectra to the red.

**TABLE I.** Absorption and emission maxima of MPTS with solvent parameters.

Solvent	λ_abs_ (nm)	λ_em_ (nm)	Stokes shift (eV)	$\pi^{*}$^a^	α^a^	β^a^	Relative polarity^b^	Viscosity (cP)^c^
Water	404	430	0.1856	1.09	1.17	0.47	1.000	1.00
Methanol	400	412	0.0903	0.60	0.98	0.66	0.762	0.55
DMSO	398	407	0.0689	1.00	0.00	0.76	0.444	2.00

^a^ These values were taken from the Kamlet–Taft solvent parameters^34,35^ where $\pi^{*}$ represents the non-specific dipolarity/polarizability, α represents the H-bond donor strength (i.e., acidity), and β represents the H-bond acceptor strength (i.e., basicity) of the solvent.

^b^ The normalized $E_{T}^{N}$ value as an empirical one-parameter scale to characterize the solvent polarity (a high value corresponds to high solvent polarity).^35^

^c^ Viscosity was measured in units of centipoise (cP, 1 cP = 1 mPa·s) at 20 °C.

The solvatochromism is also apparent in fluorescence measurements, where the emission peak wavelength is reddest in water (430 nm) and bluest in DMSO (407 nm). The Stokes shift for MPTS in water is more than twice as large as in methanol (Table I), which implies an intramolecular charge-transfer (ICT) state from the MPTS donor to acceptor sites following photoexcitation. In accordance with the observed correlation between emission λ_max_ and solvent polarity (Table I), ICT in S_1_ leads to an increased dipole moment with respect to S_0_.^24^ In other words, a solvent with higher polarity can better interact with the polar solute in the excited state and lead to a more stabilized S_1_ than S_0_ state, hence the redshift of electronic bands (Fig. 1). The ICT states have been reported in similar systems including HPTS,^8,11,18,36,37^ where the photoinduced electron redistribution leads to a cumulative separation of charge and activation of the ESPT reaction. Notably, Gaussian calculations of electron density maps of the frontier molecular orbitals (see Fig. S1 in the supplementary material) support the ICT character in the Franck–Condon region. The HOMO displays a relatively evenly distributed electron density across the conjugated ring system. In contrast, the LUMO shows a charge migration from the –OCH_3_ group to the core aromatic ring structure and three –SO_3_^−^ groups, consistent with the electron donating or withdrawing strengths of the respective substituents. A methoxy group is deemed relatively neutral though it typically displays electron-donating properties due to the oxygen's lone pair, while the sulfonate ion is considered a strong electron-withdrawing group.^18,19^ Together, a push-pull effect enables the ICT character of the photoexcited chromophore,^23,33,38,39^ which likely proceeds as the molecule becomes better solvated and energetically relaxes in the electronic excited state. In addition, both the absorption and emission spectra are much broader in water relative to methanol and DMSO.^9,11^ This result indicates more inhomogeneity in aqueous solution, likely due to the smaller size of water molecules and stronger H-bonding ability that allow for more solute conformations.

### B. Transient absorption (TA) spectroscopy of MPTS in solution

To evaluate the excited-state dynamics of MPTS and guide the wavelength selection for FSRS,^16^ we performed fs-TA experiments with 400 nm excitation to directly pump the S_0_ → S_1_ transition in all three solvents and resolved the MPTS electronic dynamics with fs resolution after photoexcitation. Starting from time zero, two prominent features last the duration of the TA data: a negative stimulated emission (SE) band from ca. 425–475 nm and a positive excited state absorption (ESA) band from ca. 500–625 nm. The SE band, similar in wavelength to the steady-state fluorescence (Fig. 1), represents the downward S_1_ → S_0_ transition while the ESA represents the upward S_1_ → S_n_ transition. Essentially, both electronic features characterize the S_1_ population.

The ESA band appears to be the broadest in water, consistent with the steady-state analysis (Fig. 1). Upon further inspection, both the SE and ESA bands exhibit center-wavelength shifts (*vide infra*) besides intensity decay mainly on the picosecond timescales (Fig. 3), in accordance with the S_1_ state energy relaxation.^20,21,40^ In particular, MPTS shows similar blueshift magnitudes of ∼17 and 15 nm in water and methanol. Using the energy unit, water leads to a notably larger relaxation (0.071 eV) than MeOH (0.055 eV) or DMSO (0.018 eV). The solvation process thus involves a relaxation from the Franck–Condon region to an adjacent CT state, and the S_1_ relaxation magnitude correlates with the solvent polarity and H-bond donating ability (Table I).

To better resolve the underlying dynamics from a blueshifting ESA band that overlaps with the SE band on the blue side, global analysis was employed (Fig. S2) to confirm the time constants obtained by integrating the entire ESA feature (Fig. 3).^41^ Three time constants representing the solvation, rotation, and emission events were necessary to sufficiently fit the data in all three solvents. The fastest solvation occurs in water (∼1.0 ps), followed by DMSO (∼3.0 ps), and MeOH (∼5.0 ps). These values largely match the average solvation time constants previously reported.^42^ Interestingly, most of the population remains in S_1_ during solvation as the ESA band does not decay significantly on this timescale, reflected in the least squares fit where the solvation component amplitude is only 6% in water [see Fig. S5(a)], yet still larger than in methanol (3%) and DMSO (2%). In addition to the wavelength shift magnitude previously discussed, this result provides further evidence of better solvation of MPTS by water due to enhanced H-bonding ability (i.e., water molecules act as the H-bond donors in this case). Furthermore, the small and facile water molecules may have the versatility to reorient and accommodate the “hot” solute molecule following actinic photoexcitation whereas the bulkier DMSO and MeOH struggle to some extent to rapidly adapt within the first solvation shell.^10,11,43^

The second time constant represents the rotational relaxation of the solute. The timescale for rotation can be estimated by the Debye theory,^24,44,45^ assuming the molecule as a sphere

$$\tau_{\mathrm{rot}}=\frac{1}{6D_{\mathrm{rot}}}=\frac{\eta V}{k_{B}T}.$$ (1)

Several factors involved in the rotational time include the volume of the spherical molecule (V), solvent viscosity (η), Boltzmann constant ($k_{B}$), and temperature (T). The volume of MPTS remains roughly constant regardless of the solvent, but an interesting size comparison with HPTS can be made. The rotational relaxation of HPTS at pH = 12 and 4.5, where the molecule is, respectively, deprotonated and protonated at equilibrium, occurs with the time constants of 83 and 90 ps in water.^11,21,40^ For MPTS in water, the rotational relaxation proceeds with a slightly longer time constant of 98 ± 4.0 ps without the contribution from ion-pair separation as in HPTS.^10,11,21^ This analysis agrees with theory as the solute size increases due to the one side chain changing from –O^−^, –OH, to –OCH_3_, the rotational relaxation slows down. Since methanol has the lowest viscosity (0.55 cP, Table I), the TA intensity dynamics show the fastest rotational time constant of 90 ± 4.5 ps in Fig. 3. Correspondingly, DMSO has the longest time constant of 210 ± 5.0 ps because it is significantly more viscous (2.00 cP, Table I).

The Gaussian-optimized molecular structure of MPTS has an estimated sphere radius of 4.6 Å along the long vertical axis.^18,29^ Using Eq. (1) at room temperature (20 °C to match Table I), the $\tau_{\mathrm{rot}}$ is predicted to be 101, 55, and 202 ps in water, methanol, and DMSO, respectively. The rotational time constants of MPTS in water and DMSO (98 ps and 210 ps) are consistent with these estimates, where DMSO is twice as viscous (see Table I). In contrast, MPTS in methanol displays a much longer rotational time constant (90 ps) than expected due to viscosity alone (55 ps). A similar observation was made in fluorescence upconversion experiments of HPTS in methanol: the counterion association between the Na^+^ and SO_3_^−^ ions significantly increases the volume of the rotating solute by ∼3.1 times and lengthens the rotational/orientational-relaxation time constant to ∼190 ps.^12^ In contrast, no counterion association was observed in our fs-TA experiments, evidenced by the much faster 90 ps rotational time constant for the photoexcited MPTS in methanol (Fig. 3), though an ∼18% increase in the molecular radius is necessary for the predicted rotational time to match the experimental value of 90 ps. This is reasonable considering the fact that the solute–solvent H-bonding complex is larger than the solute itself,^12^ but such size corrections may have a solvent-dependent nature.

To further confirm the assignment of this rotational relaxation component, control experiments were performed in the H_2_O:glycerol and MeOH:glycerol binary solvent mixture (50% v:v) with steady-state electronic spectra (Fig. S3) and fs-TA spectra (Fig. S4). With the significantly increased viscosity of glycerol, the rotational relaxation of the solvated MPTS chromophore would lengthen drastically. For example, the viscosity of a 50% v:v (∼55% w:w) H_2_O:glycerol solution is ∼8.00 cP at 20 °C.^46^ According to Debye theory [Eq. (1)], the $\tau_{\mathrm{rot}}$ in the mixture should be ∼8 times of pure water (98 ps in Fig. 3) reaching 784 ps, while the observed value is ∼550 ps in Figs. S5(a) and S5(c). The difference could be a consequence of other competing nonradiative pathways in a realistic binary solvent environment, as well as the experimental uncertainty while transferring and measuring the exact volume of highly viscous glycerol to make the resultant solvent mixture.

The third time constant is mainly attributed to a fluorescence pathway as it occurs on the nanosecond timescale, characteristic of the fluorescence lifetime.^8,11,37^ However, it does not necessarily represent a pure fluorescent state because it involves an ensemble average of radiative and non-radiative processes.^24,40^ Notably, MPTS in water has the longest time constant of 2.9 ± 0.3 ns while DMSO has the shortest (1.9 ± 0.2 ns) with methanol lying in between (2.5 ± 0.2 ns). These values will be discussed further in the context of the FQY measurements (see Sec. II C below, and Fig. S6 for experimental data plots) while the information about the associated error bars can be found in the supplementary material.

The SE intensity dynamics in Fig. 3(b) is reminiscent of the ESA intensity dynamics in Fig. 3(a), except for small changes in the fluorescent lifetimes from the exponential fits. Further inspection yields several interesting results. Instead of an intensity decay as ESA, the SE intensity rises during solvation, indicative of the formation of an ICT state.^40^ Notably, ICT (a subset of CT) states are known to be sensitive to solvation and often assigned by a telltale rise of the SE band. A useful comparison can be made to HPTS in water at pH = 12,^40^ where HPTS remains predominantly in the deprotonated form, increasing the electron donating strength from the –O^−^ site and further enhancing the ICT state formation while ESPT is inhibited. Therefore, in our fs-TA spectra of the photoexcited HPTS and MTPS in aqueous solution, MPTS only shows a SE intensity rise by ∼10% during solvation while the deprotonated HPTS shows an SE intensity rise by ∼50%.^40^ Nonetheless, the SE band rises in intensity on the solvation timescale, representing an increased oscillator strength for the downward transition following the locally excited (LE) → CT transition.

### C. Fluorescence quantum yield (FQY) of MPTS in solution

To elucidate the molecular fate after arrival at the CT state, the observed TA dynamics needs to be viewed through the lens of FQY measurements. By plotting the integrated fluorescence (upon 400 nm excitation) as a function of the absorbance for dilute solutions of MPTS in the respective solvents, the slopes can be compared to those of a known standard (Coumarin-102, FQY = 0.764) so the FQY can be calculated using the following equation:^47^

$$\Phi_{f}^{i}=\Phi_{f}^{s}\frac{f_{s}(\lambda_{ex})}{f_{i}(\lambda_{ex})}\frac{\int_{\lambda_{em,1}}^{\lambda_{em,2}}F^{i}(\lambda_{em})}{\int_{\lambda_{em,1}}^{\lambda_{em,2}}F^{s}(\lambda_{em})}\frac{n_{i}^{2}}{n_{s}^{2}}.$$ (2)

The $\Phi_{f}^{x}$ represents the FQY of the sample (x=i) and known standard (x=s). The term $f_{x}\left(\lambda_{ex}\right)=1-10^{-A_{x}(\lambda_{ex})}$, where $A_{x}(\lambda_{ex})$ represents the absorbance (i.e., OD that is unitless) at the excitation wavelength. The integral $\int_{\lambda_{em,1}}^{\lambda_{em,2}}F^{x}(\lambda_{em})$ represents the integrated area of the fluorescence band within a suitable range, which was selected from 430 to 530 nm upon 400 nm excitation in this work (see the MPTS emission wavelength range in Fig. 1). Finally, the term $n_{x}^{2}$ represents the refractive index of the solvent.

Our systematic FQY measurements of the solute MPTS (Fig. S6) resulted in a consistent trend observed throughout this investigation with the solvent H-bond donating property: water has the highest FQY at 0.83, while methanol and DMSO have significantly lower values at 0.47 and 0.32, respectively. Intriguingly, these values of MPTS are lower than those of HPTS in water (0.86) and methanol (0.79).^48,49^ In water, since HPTS undergoes an ESPT reaction (i.e., PA^*^ → PB^*^) to fluoresce from a lower-lying electronic state, it likely involves more complex relaxation pathways with potential to go awry than MPTS trapped within the PA^*^ state without an ESPT coordinate. The observed higher FQY of HPTS in water can thus be attributed to a highly efficient ESPT reaction^10,11,50^ and vibrational cooling process through the phenolic hydroxy end,^51^ whereas the vibrational cooling within a trapped PA^*^ state is less effective for MPTS so other nonradiative relaxation pathways become more competitive with the radiative (fluorescence) pathway.^11,18^ Furthermore, the large difference (∼40%) of FQY values between HPTS and MPTS in methanol is interesting because both chromophores cannot undergo ESPT. A similar difference of ∼43% was observed between MPTS in water and methanol (both polar and protic solvents), indicating that the solvent exerts a significant effect on the solute energy dissipation. Notably, water and methanol have stronger H-bonding interactions with the hydroxy group of HPTS in comparison to the methoxy group of MPTS, responsible for a higher FQY of HPTS, whereas DMSO has the least H-bond donating ability and shows the lowest FQY for MPTS. These results demonstrate a consistent trend correlating the solute–solvent H-bonding strength to the energy dissipation pathways of these pyrene derivatives: the stronger the intermolecular H-bonds, the higher the FQY.

### D. Correlation between the TA dynamics and FQY of MPTS in solution

A connection between the FQY measurements and the excited-state electronic dynamics can be made in the three solvents. We first consider the fluorescence lifetime that is estimated from the longest component of TA dynamics. In water, most of the excited-state populations reach a fluorescent state, supported by the large FQY and reflected by the emission lifetime of 2.8 ns being the longest among three solvents [Fig. 3(a)]. Considering the involvement of some nonradiative relaxation pathways, the true fluorescent lifetime is likely above 3.0 ns.^6,8,52^ For MPTS in methanol with FQY = 0.47 [Fig. S6(c)], ∼50% of the ensemble chromophore populations emit fluorescence, leading to a shorter time constant of 2.5 ns due to other competing nonradiative pathways. With a further reduced FQY = 0.32 [Fig. S6(d)], DMSO shows the shortest time constant of 2.0 ns on this mainly emission stage. This trend is more apparent in the SE dynamics, where water and DMSO have even longer and shorter lifetimes on the nanosecond timescale, respectively [Fig. 3(b)].

Notably during the intermediate rotation stage, nuclear motions of the MPTS chromophore could thermally release energy to the solvent and involve a nonradiative transition back to the ground state (S_0_). This may occur to some extent because the ESA and SE bands significantly decay on this timescale; however, it seems unreasonable for a relatively low-energy process like molecular rotation to release enough energy to return most of the populations to S_0_. Instead, the rotational relaxation more likely involves a cooling process within the electronic excited state (e.g., S_1_) of the solute-solvent complex. For instance, MPTS in water has the highest FQY and longest emission lifetime, implying that a majority of the chromophore population reaches a fluorescent state. Among the three solvents, the largest amplitude of the rotational component occurs in water, consistent with its strongest H-bonding interaction with MPTS. In particular, the ESA intensity dynamics show the amplitude weights of 32%, 23%, and 14% in water, methanol, and DMSO (Fig. S5), while the SE intensity dynamics manifests even greater separation of the weights (48%, 30%, and 17%). If this rotation stage predominantly involved a nonradiative transition back to S_0_, the FQY measurements would have displayed an opposite trend with the highest FQY in DMSO and the lowest in water. In contrast, the rotational motion could essentially help the photoexcited MPTS population reach a state that has a greater propensity to fluoresce. Further evidence comes from fs-TA spectral profiles from global analysis (Fig. S2). We cannot claim that all the rotational motions lead to a highly fluorescent state because it is plausible that a portion of the rotational relaxation enables a nonradiative transition back to the electronic ground state.^23,24,40^

Solvation plays a pivotal role in leading to a fluorescent state, yet has a minimal effect on the TA intensity dynamics (see Figs. 3 and S5). The solvation effect can be examined from the center wavelength (λ_max_) shift of the ESA and SE bands as a function of time, a reliable observable that is not affected by fluctuations in laser power or sample concentration.^20^ In all three solvents (Fig. 4), a biexponential fit of the dynamics is sufficient; notably, the average of the two fitted time constants closely matches the solvation time obtained from the intensity dynamics: ∼1, 3, and 5 ps (Fig. 3) for MPTS in water, DMSO, and methanol, respectively. The sub-picosecond component from the λ_max_ blueshift likely reflects some hindered small-scale solvent motions,^53,54^ involving local reorientation of solvent molecules (e.g., water librations) to rapidly accommodate the photoexcited MPTS solute molecules.^18,55,56^ The longer picosecond component likely represents the typical solvent reorganization termed the longitudinal relaxation, which is a more coordinated process between the solute and the first few solvation shells.^11,57,58^ Interestingly, the sub-picosecond component may be faster in DMSO (than MeOH) due to the lack of H-bonding between solvent molecules^59^ that allows the initial response to be swift. In contrast, the more H-bonded methanol molecules have a relatively slow process within the first solvation shell.^30^ Water has even stronger H-bonds, while the smaller molecular size and unique H-bonding network enable an ultrafast response to the photoexcited molecule [Fig. 4(c)].

**FIG. 2.**
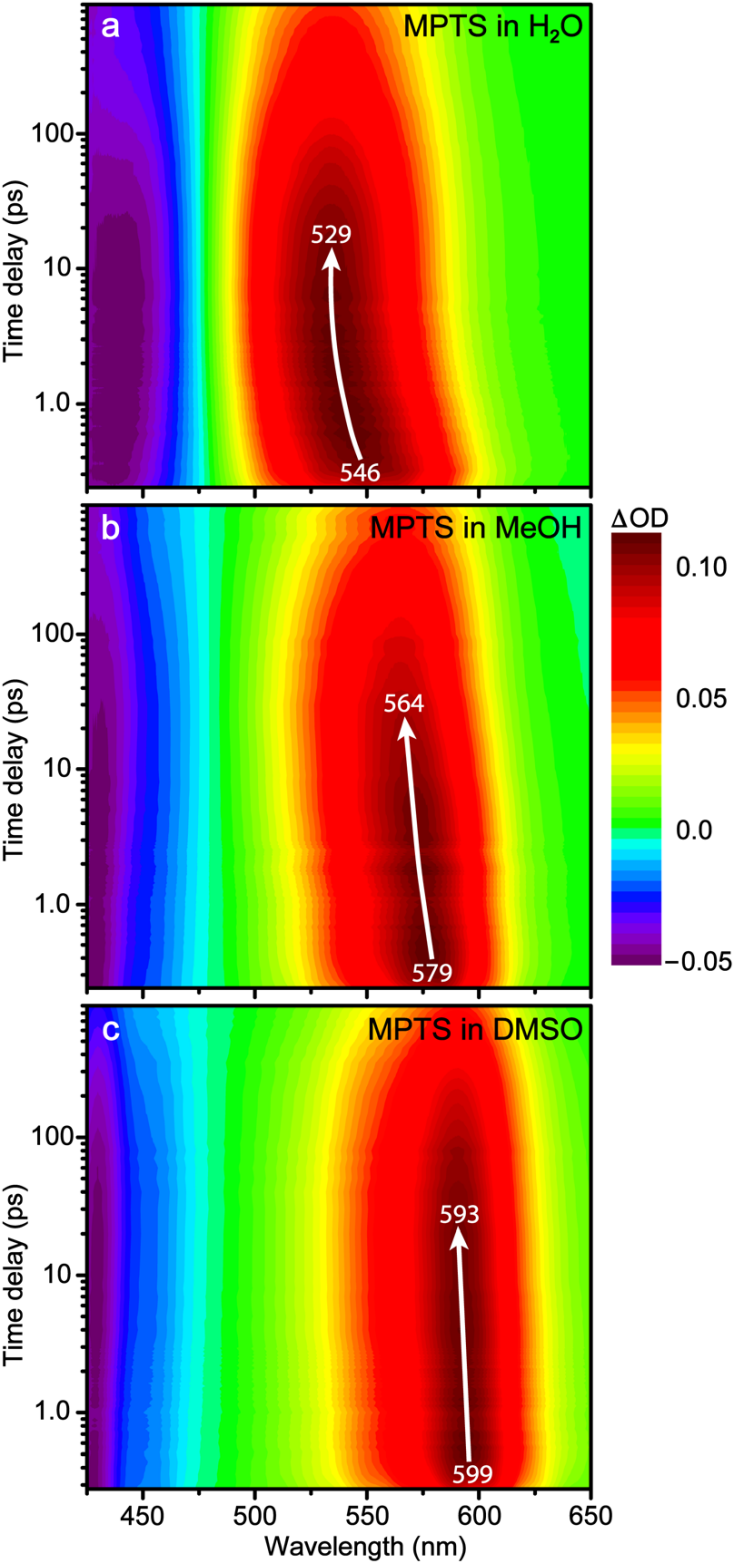
Semi-logarithmic contour plots of the fs-TA spectra upon 400 nm excitation of MPTS in (a) water, (b) methanol, and (c) dimethyl sulfoxide. The color-coded intensity levels (shown to the right) apply to all three plots. The white arrow traces the blueshift of the excited state absorption (ESA) band with its initial and final center wavelengths listed within the collection time window.

**FIG. 3.**
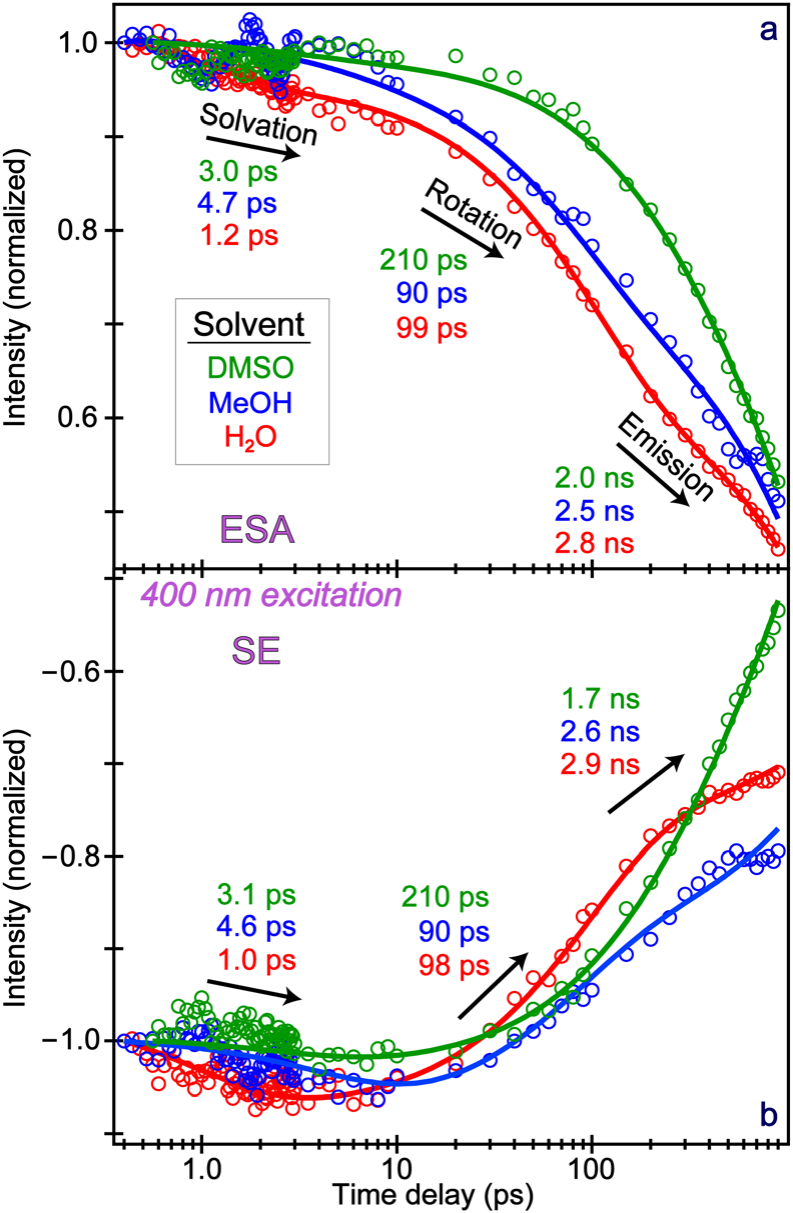
The integrated intensity of the (a) ESA and (b) SE bands from fs-TA experiments with 400 nm excitation in DMSO (green), MeOH (blue), and H_2_O (red) plotted on the logarithmic timescale. The tri-exponential least squares fits are shown as the color-coded solid curves overlaid with data points (circles), and the retrieved average time constants are listed by the respective arrows corresponding to characteristic processes on ultrafast timescales.

If the ESA and SE dynamics report on similar S_1_ populations, an ESA blueshift should be accompanied by an SE redshift. A detailed analysis of the blue-wavelength region (<475 nm) in Fig. 2 reveals such a redshift (Fig. S7). Though the qualitative trends are consistent, the SE redshift dynamics are faster than the ESA blueshift. This could be owing to the spectral overlap from the ground state bleach on the bluer side and a stronger ESA band on the redder side. The control experiments with glycerol (Fig. S8) significantly slow down the ESA and SE shifts, indicative of a damped solvent response and the subsequent lengthening of solvation time constants. The cumulative shift of ESA and SE bands in tandem substantiates the solvation process accompanying an ultrafast transition from the LE to CT state while undergoing energy relaxation. A notable portion of the S_1_ population remains on this timescale as shown by the intensity dynamics (Fig. 3); this is likely a preparatory step that undergoes rotational relaxation to reach a fluorescent state.^24^ We also note that the largely unchanged transition dipole moment of MPTS in solution for the radiative emission allows the tracking of time-resolved fluorescence anisotropy undergoing an exponential decay as reported,^12,25^ which is determined by the rotational diffusion of a molecule.

### E. Femtosecond stimulated Raman spectroscopy (FSRS) of MPTS in solution

**FIG. 4.**
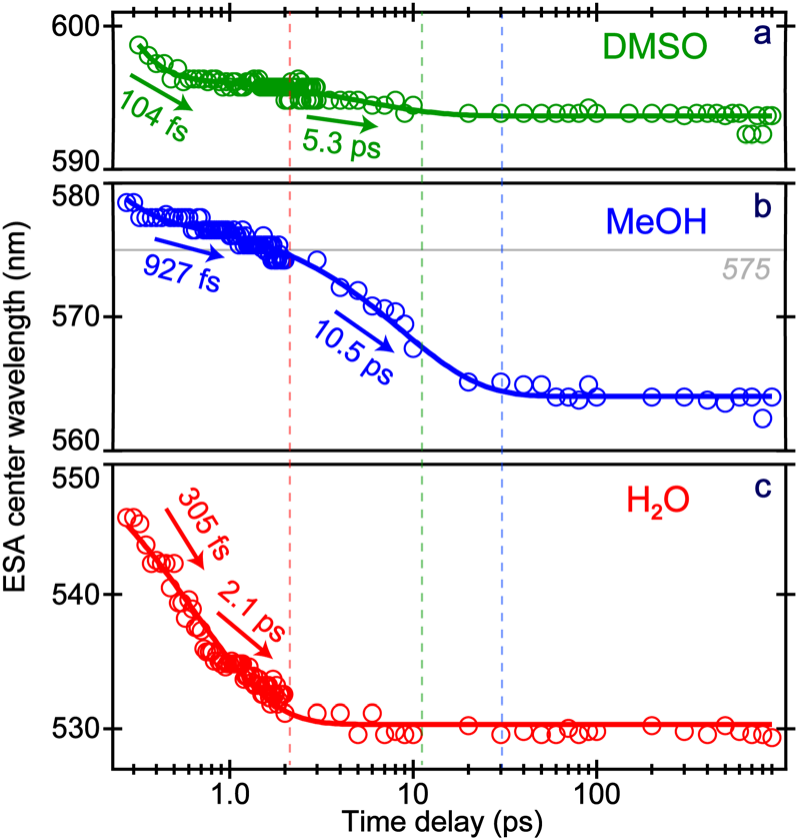
The center wavelength (nm) blueshift of the ESA band of MPTS following 400 nm excitation in (a) DMSO (green), (b) MeOH (blue), and (c) H_2_O (red) with a logarithmic time axis. The color-coded dashed vertical lines indicate when the shift essentially ends in the respective solvents. The least squares biexponential fits are shown as solid curved lines with time constants listed by the respective arrows. The Raman pump at 575 nm used in FSRS experiments is marked.

The fs-TA results reveal the ultrafast electronic dynamics with major steps in S_1_, though little information is available regarding the pertinent atomic motions.^16,23^ To provide such structural dynamics information, time-resolved FSRS was performed on MPTS in H_2_O and MeOH following photoexcitation. FSRS allows for a glimpse into the vibrational motions that play active roles and govern the excited-state energy dissipation with sufficient temporal and spectral resolutions.^16,23,28^ Identical to TA experiments, the 400 nm actinic pump was used. The Raman pump was tuned to 575 nm to be resonant with the ESA band, hence significantly enhancing the excited-state FSRS signal with absorptive (positive) Raman peaks.^11,49^ This pump position remains on the red side of the ESA peak (see Fig. 2) throughout the experimental time window in water. However, in methanol the stationary Raman pump begins on the blue side of the ESA peak at time zero and becomes more on resonance as the ESA band blueshifts, eventually achieving pre-resonance on the red side of the ESA band at later times [see Fig. 4(b)]. The influence of the Raman pump position has been discussed in previous papers^23,28,49,51,60,61^ and is still an ongoing area of research.

**FIG. 5.**
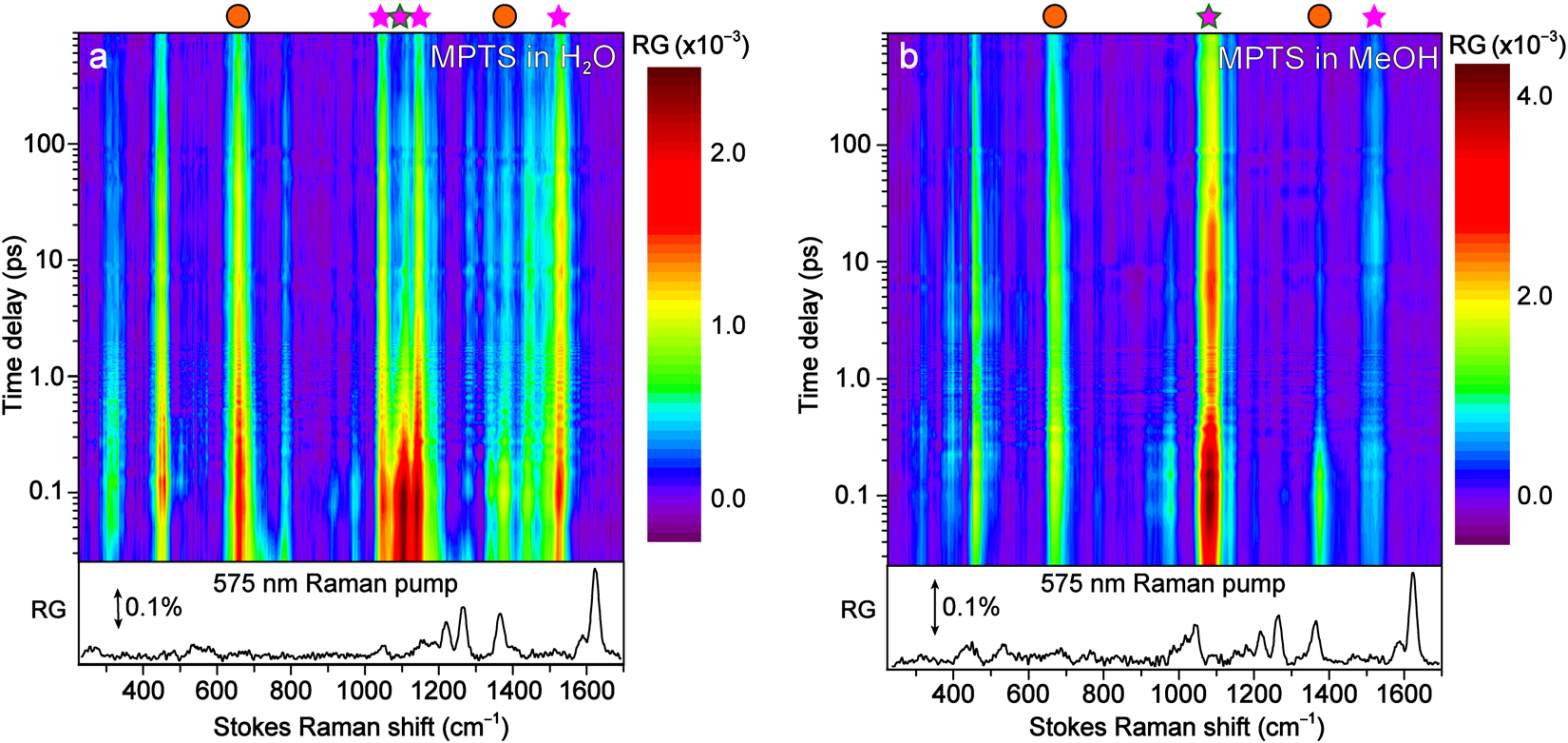
The semi-logarithmic 2D contour plot of the excited-state FSRS spectra upon 400 nm excitation of MPTS in (a) H_2_O and (b) MeOH. The ground-state FSRS spectra are shown below the respective plots. The Raman pump was tuned to 575 nm to be within the ESA band and achieve resonance enhancement, the probe window is ca. 250–1700 cm^−1^ on the Stokes side. The stimulated Raman gain (RG) intensity color bar is displayed to the right of the time-resolved plot. The double-sided arrows by the ground-state spectra indicate a RG magnitude of 0.1%. Prominent peaks are highlighted by various symbols above: stars, analyzed in main text; circles, detailed in the supplementary material with additional vibrational analysis.

The ground-state FSRS data are similar in both solvents; most of the intense peaks appear at the high-frequency side between ∼1000 and 1650 cm^−1^. These experimental peak positions and relative intensities were verified by quantum calculations of MPTS in both solvents (see methods in the supplementary material), implying that the solvent does not have a significant effect on the vibrational motions of MPTS at equilibrium. A pronounced high-frequency peak is observed at ∼1525 cm^−1^, which is red-shifted from its ground-state counterpart (see Fig. 5). This peak was assigned to an asymmetric C = C stretching motion along the vertical axis of the MPTS conjugated ring system.^11,18^ The redshift of this mode from S_0_ to S_1_ supports the formation of an ICT state following prompt electronic redistribution and can be visualized via the frontier molecular orbitals (Fig. S1): the electron density shifts from being aligned along the vertical C = C (through-atom axis) to the horizontal C = C bonds (through-bond axis) on the central conjugated rings.

**FIG. 6.**
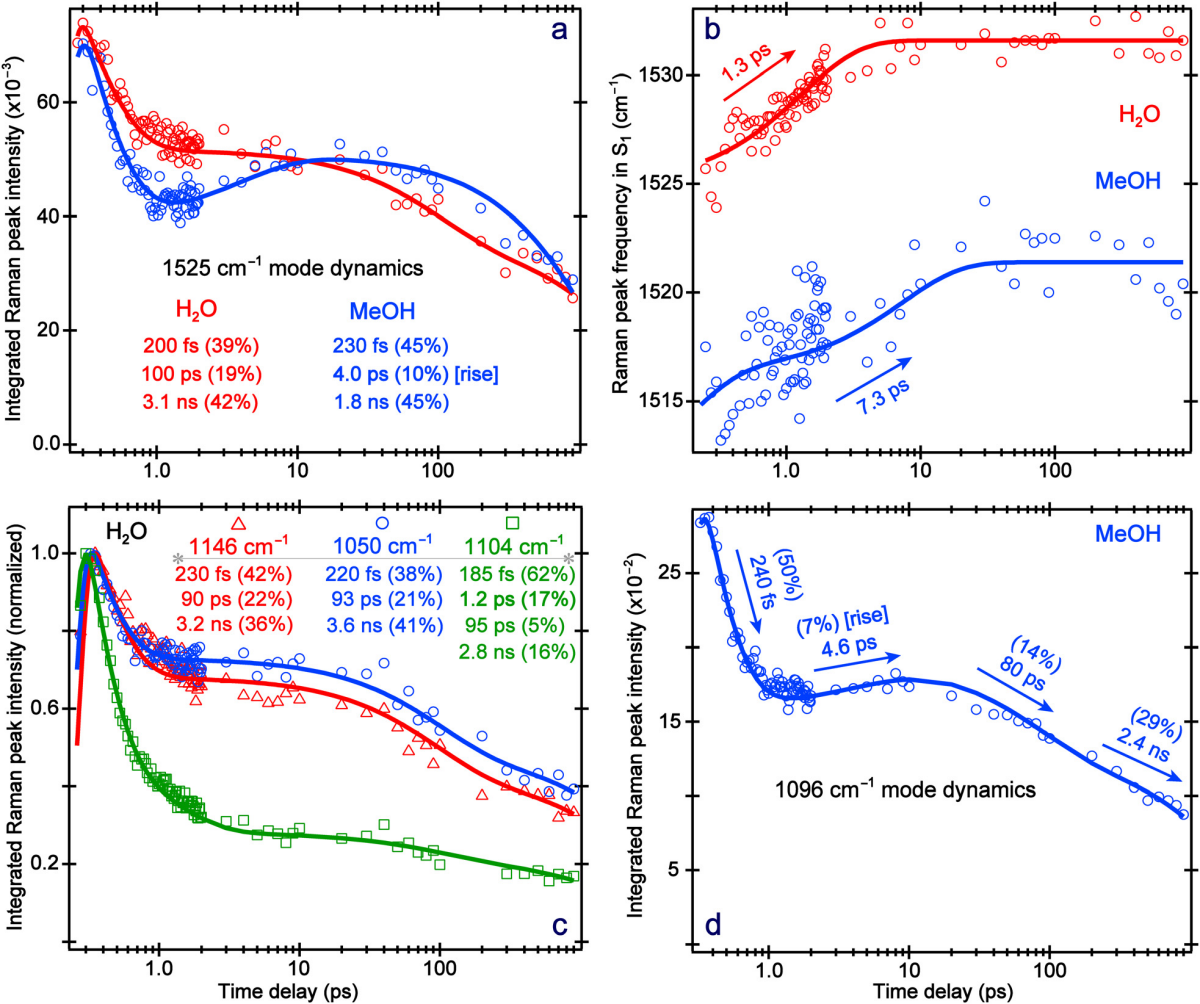
Intensity and frequency dynamics of the excited-state vibrational peaks from time-resolved FSRS experiments on MPTS in water and methanol after 400 nm excitation. (a) Intensity dynamics of the ∼1525 cm^−1^ mode in water (red) and methanol (blue). (b) Frequency dynamics of the ∼1525 cm^−1^ mode in water (red) and methanol (blue). (c) Normalized intensity dynamics of the triplet of peaks at 1146 cm^−1^ (red triangles), 1104 cm^−1^ (green squares), and 1050 cm^−1^ (blue circles) in water. (d) Intensity dynamics of the 1096 cm^−1^ mode in methanol. The time axis is shown on a logarithmic scale. The least squares fits of spectral data dynamics are shown as the color-coded solid curves. The retrieved average time constants and amplitude weight percentages are listed as insets.

Notably, this vibrational marker band center frequency exhibits a blueshift in both water and methanol [Fig. 6(b)] on the solvation timescale. The blueshift from 1525 → 1532 cm^−1^ has a 1.3 ± 0.2 ps time constant in water, and from 1515 → 1522 cm^−1^ with a 7.3 ± 0.4 ps time constant in methanol, characteristic of vibrational cooling^51,62,63^ toward a lower portion of the trapped S_1_ state of MPTS. On these picosecond timescales reminiscent of the solvation time constant from TA dynamics (see Fig. 3), the “hot” photoexcited molecules release energy as solvation proceeds, while the PES anharmonicity results in an increase in vibrational energy level spacing.^51,62,63^ A similar cooling-induced blueshift was observed for the ∼1380 cm^−1^ mode in water but not in methanol [Fig. S9(b)], since the mode frequency dynamics can also be affected by the nonequilibrium conformational motions of the solute molecule that may counteract the blueshift factor.^13,20^

The excited-state FSRS peak intensity dynamics of MPTS (Fig. 5) displays several similarities and differences when compared to the fs-TA dynamics in water and methanol (Fig. 3). First, the initial solvation time constant of 1–5 ps is replaced with a much faster ∼200 fs time constant in both solvents [Fig. 6(a)]. This is reasonable since the Franck–Condon dynamics on the hundreds of femtoseconds timescale is commonly observed in FSRS data following photoexcitation due to a swift change of resonance Raman conditions.^11,23^ This high-frequency C = C stretching motion may be responsible for an effective energy transfer from the methoxy group through the core of the conjugated rings to the sulfonate groups during initial solvation; hence, the significant change of electric polarizability and the observed intensity drop within ∼1 ps in Fig. 6(a).^50,64^

Second, the next two components from FSRS mode intensity dynamics closely match the TA intensity dynamics in water. The ∼95 ps component represents the rotational relaxation, and the chromophore vibrational modes could play a role in this process. The ∼3.1 ns component mainly reflects the fluorescence lifetime, matching the TA result (2.8–2.9 ns, Fig. 3). In contrast, besides a ∼1.8 ns decay, the ∼1520 cm^−1^ peak evolution in methanol shows a rise component of 4.0 ± 0.5 ps that matches the average solvation time of methanol.^20,42,49^ This observation could stem from the resonance conditions experienced by a stationary Raman pump (i.e., fixed at 575 nm during the excited-state FSRS). The ESA band shifts with an average time constant of ∼5 ps from 580 → 565 nm [Figs. 4(b) and S2(b)], achieving dynamic resonance conditions^16,23,49,64^ with the 575 nm Raman pump, hence, a ∼4 ps rise in FSRS mode intensity dynamics. In addition, as the photoexcited MPTS becomes solvated in a H-bonding network, the polarizability of specific nuclear motions could increase in a mode-dependent manner (e.g., enlarging the molecular volume),^16,24^ hence the observed Raman gain increase.

Importantly, nearly all the excited-state Raman modes of MPTS in methanol show similar rise dynamics between ca. 1 and 20 ps [see Fig. 5(b)], whereas a similar phenomenon is not observed in water [Fig. 5(a)]. To corroborate the aforementioned dynamic resonance effect^16,23,49^ as a major contributor to this intermediate rise component in methanol, we performed control experiments by tuning the Raman pump to redder wavelengths at 590 and 600 nm. This setup would essentially replicate the MPTS in the water experiment wherein the Raman pump starts on the red edge of the ESA band from time zero. Indeed, these redder Raman pump wavelengths resulted in pure decay dynamics, like the red curve in Fig. 6(a) for MPTS in water. However, the use of a redder Raman pump results in a diminished signal-to-noise ratio, especially at later times when the blue-shifted ESA band becomes completely off-resonant. This issue was not experienced during the FSRS experiments of MPTS in water, likely due to the broadness of the ESA band [see Fig. 2(a)].

**FIG. 7.**
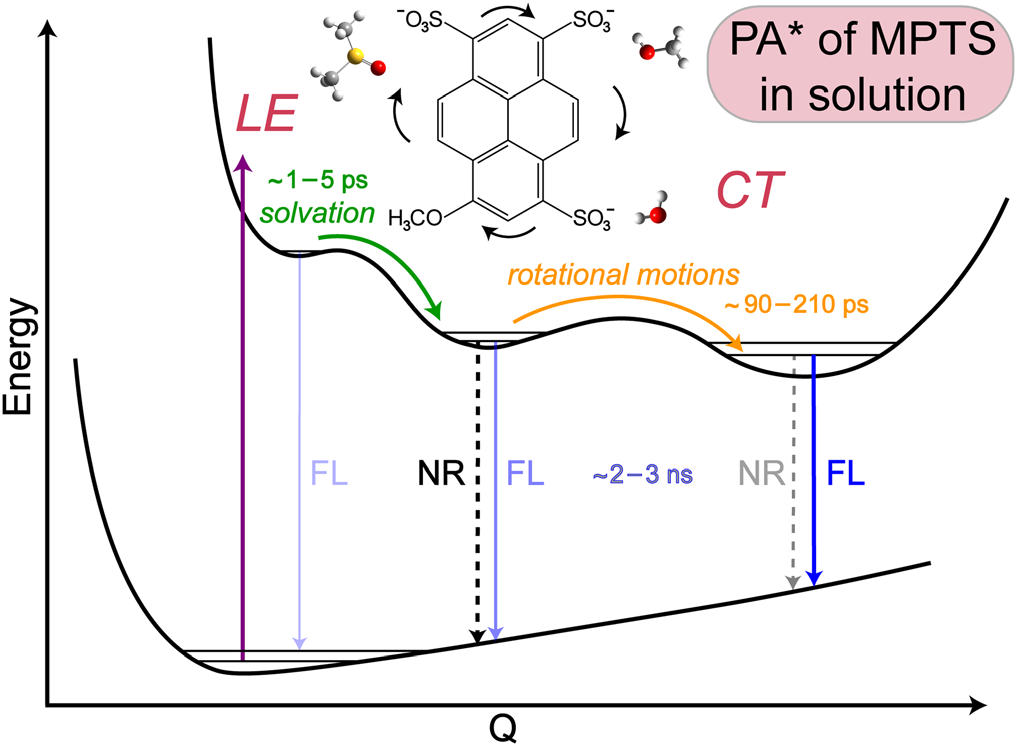
Proposed potential energy surface of the photoexcited MPTS chromophores in solution. After vertical excitation into the locally excited (LE) state, the nascent charge-transfer (CT) state is stabilized by a solvation step (green arrow). The excited-state molecules could undergo the nonradiative (NR) and radiative (fluorescence, or FL) relaxations to varying degrees (bolder arrows mean stronger electronic transitions) before and after the small rotational barrier (beneath the orange arrow). The characteristic timescales for the depicted excited-state relaxation processes are listed. The MPTS chemical structure with three different solvent molecules is shown (inset) with the rotational motions within the CT state illustrated by the black curved arrows.

The fluorescent lifetime provides mechanistic insights into the fate of the rotational relaxation. Consistent with the trend observed in fs-TA dynamics, the nanosecond component from FSRS data in Fig. 6(a) is much faster in methanol (1.8 ± 0.3 ns) than in water (3.1 ± 0.2 ns), implying an enhanced nonradiative pathway in the former solvent. Noticeably lacking a prominent rotational component in methanol, this ∼1520 cm^−1^ mode may not actively participate in the crossing of the rotational barrier to a lower-lying fluorescent state. This motion could occur primarily within a trapped region of the excited state with the CT character and enhanced nonradiative pathways, leading to an apparently reduced nanosecond lifetime. In Figs. 6(a) and S9(a), Raman marker bands that show a rotational component also exhibit a longer emission lifetime, consistent with our proposed model that the rotational relaxation leads to a more fluorescent state (darkest blue arrow in Fig. 7) while the steady-state fluorescence spectrum is the broadest in water (Fig. 1). In fact, most of the Raman modes in water manifest a rotational component, whereas only a select few are observed in methanol. Therefore, these characteristic vibrations could act as sensitive probes for rotational relaxation and/or diffusion, which involves an intricate interplay between the solute and solvent. As fewer modes of MPTS track the rotational dynamics in methanol with an estimated increase in rotating volume by ∼64% (see Sec. II B) to match the observed time constant, a markedly higher rotational barrier (than in water) leads to less excited-state populations reaching the more fluorescent state, corroborated by the much smaller FQY of MPTS in methanol (0.47) than in water (0.83) (see Fig. S6).

For the photoexcited MPTS in solution, several conserved Raman modes are present between ca. 1200 and 1500 cm^−1^ involving similar motions (C = C stretch) and dynamics (Fig. 5). On the lower energy side, a triplet of peaks from ∼1050–1150 cm^−1^ in water [Fig. 5(a)] display quite different decay dynamics than the single dominant peak around 1096 cm^−1^ in methanol [Fig. 5(b)]. The nearby C–OH stretching motion of solvent methanol at ∼1037 cm^−1^ causes some bleaching; however, a careful solvent addback procedure mitigated this issue by giving a largely solvent-free spectrum at each time point.^20,49^ Moreover, independent data processing without solvent addback provided similar solute dynamics, confirming that the transient solvent signal does not obfuscate the analysis. In methanol, the first two components are similar to the ∼1520 cm^−1^ mode and can be interpreted similarly [Fig. 6(d)]. An additional 80 ± 3.0 ps time constant is necessary to fit the dynamics with a 14% amplitude weight, notably less than the rotational amplitude of the ∼1530 (19%), 1146 (22%), and 1050 (21%) cm^−1^ peaks in water [Figs. 6(a) and 6(c)]. This trend is also present in the fs-TA experiments, where the rotational component is more prominent in the intensity decay of the ESA and SE bands in water (Figs. 3 and S5). On the other hand, when comparing the ∼1520 and 1096 cm^−1^ peaks in methanol in Figs. 6(a) and 6(d), the latter mode shows a longer emission lifetime by ∼0.6 ns. This enlarged time constant reflects the increased probability to reach a more fluorescent state by undergoing rotational relaxation, likely facilitated by this 1096 cm^−1^ mode. The intensity dynamics of other Raman marker bands [∼1380 cm^−1^ mode in Fig. S9(a) and 660 cm^−1^ mode in Fig. S11(a)] supports this interpretation, and substantiates the mode-dependent nature along the photoinduced multidimensional excited-state energy relaxation pathways (Fig. 7).

A more illuminating result can be deduced from the peak triplet in water. From the 2D contour plot in Fig. 5(a), the two outer peaks clearly display longer lifetimes than the middle peak. Indeed, this result is confirmed in Fig. 6(c), where the 1104 cm^−1^ mode decays much faster than the outer 1146 and 1050 cm^−1^ modes. Assigned to a C–SO_3_^−^ stretching motion coupled with ring in-plane deformation on the basis of density functional theory calculations and consistent with literature,^11,18^ the 1104 cm^−1^ mode is the only vibrational peak that decays on the timescale of solvation with an eminent amplitude (17%). When studying the photoacid HPTS and methoxy derivative MPTS, it has been well documented that the sulfonate groups are primarily responsible for improving the solubility in polar solvents.^11,12,18,19^ Our current finding presents additional experimental evidence for the importance of these side chain sulfonate groups in solution, especially for MPTS with reduced H-bonding properties of the methoxy group (vs a hydroxy group). In previous FSRS experiments of HPTS, a similar peak pattern and intensity decay were observed with a solvation component of 800 fs (∼20% weight),^11^ corroborating the MPTS data in water. Furthermore, the dynamics of the C–SO_3_^−^ stretch only show a weak rotational amplitude (5%) leading to a relatively fast emission component (∼2.8 ns lifetime) compared to the outer two modes (3.2–3.6 ns lifetimes). This result implies that the sulfonate groups play a prominent role during the solvation stage, yet do not actively participate in the rotational cooling. The 1146 and 1050 cm^−1^ modes manifest relatively strong rotational components (∼22% weight), so they could play a more significant role in the rotational cooling with accordingly longer fluorescent lifetimes (i.e., crossing the small rotational barrier depicted in Fig. 7 within a relatively flat PES in S_1_) and less contributions from other nonradiative pathways. These modes are mainly associated with the conjugated ring in-plane deformation with ring-H rocking motions^18,50^ that could help the collective molecular framework overcome the low barrier to a more fluorescent state.

Further experimental evidence to corroborate these findings can be found in the excited-state vibrational frequency dynamics (Fig. S10): the ∼1104 cm^−1^ mode of MPTS in water displays a dominant blueshift on the solvation timescale (1.5 ps) and a small blueshift on the rotational relaxation timescale (95 ps), while the adjacent 1050 cm^−1^ mode remains largely unshifted during the experimental time window of 900 ps. This observation substantiates the mode-dependent nature of the FSRS signal as different vibrational modes respond to the photoenergy dissipation differently in terms of electrostatics and structural coordinates, hence tracking the vibrational cooling and/or conformational dynamics (see detailed discussions after Fig. S10 in the supplementary material).^11,13^ Given the interplay between electronic and vibrational motions,^23^ a more electron-donating group (e.g., –NH_2_) instead of –OCH_3_ may enhance the ICT character of MPTS,^33^ while an electron-withdrawing group such as NO_2_ attached to an aromatic ring system could act as a molecular motor to quench fluorescence via a conical intersection.^65^

## III. CONCLUSIONS

In this work, we have investigated MPTS as an important derivative of the widely used photoacid HPTS, wherein the replacement of a single proton with a methyl group exposes the effects of methylation on the excited-state potential energy surface of the solvated chromophore. An integrated advanced spectroscopic toolset including the steady-state electronic measurements, fs-TA, and tunable FSRS in the ground and excited states, aided by quantum calculations, enables the elucidation of energy dissipation pathways of MPTS in solution following photoexcitation. Aside from the elimination of a proton transfer coordinate, several property changes are observed within the excited state manifold for the methylated photoacid derivative. The fs-TA results of MPTS in water, methanol, and DMSO, as well as control experiments by adding 50% (v:v) glycerol, reveal that solute–solvent interactions significantly dictate the energy dissipation in the S_1_ state. The blueshift of the ESA band as well as the rise and redshift of the SE band occurs on the typical solvation timescale during the LE → CT transition, fastest in water (∼1.0 ps) and slowest in methanol (∼5.0 ps). The magnitude of the solvation is directly correlated with the H-bond donating ability of the solvent because MPTS does not have prominent H-bond donating substituents. Systematic FSRS analysis and quantum calculations of an array of vibrational modes provide structural dynamics information in the excited state, where the sulfonate groups are experimentally verified to actively participate in the ultrafast solvation events in protic solvents (e.g., water, methanol). Furthermore, the methoxy group diminishes the amount of CT character across the molecular framework following 400 nm photoexcitation, which may explain why methylated molecules commonly display reduced reactivity.^11,12,18,19^

Without a proton transfer coordinate, the molecule needs to navigate the excited-state potential energy landscape to effectively dissipate the photoexcitation energy.^13,23^ Following the formation of a transient CT state, the chromophore population may remain trapped with a higher tendency to nonradiatively return to the ground state, or overcome a small rotational barrier to reach a more fluorescent state. The timescale for the rotational relaxation was estimated according to Debye theory and is consistent with the retrieved values from fs-TA and FSRS experiments. In particular, methanol displays the fastest rotational component (90 ps), followed by water (98 ps) and DMSO (210 ps). The lengthening of the rotational motion time constant corresponds with the increased solvent viscosity assuming a similar volume of the solute molecule. Reminiscent of the solvation component, the rotational relaxation becomes enhanced by the H-bond donating ability and relative polarity of the solvent, MPTS in water therefore undergoes the rotational motion to a larger degree than in the other solvents. We propose that such rotational motions could facilitate the fluorescence from an overall trapped PA^*^ electronic state (Fig. 7), supported by the FQY measurements wherein MPTS in water displays the longest emission lifetime with a significantly higher FQY value (0.83) than methanol (0.47) and DMSO (0.32). To substantiate the contributions of molecular rotational relaxation or diffusion events to the observed spectral patterns with all parallel-polarized laser pulses in this work, further control experiments can be performed with the polarization-dependent ultrafast spectroscopic techniques (e.g., upconversion fluorescence,^12^ TA, and FSRS) in correlation with advanced simulations of molecular response functions.^66–68^

In summary, our current electronic and structural dynamics findings bridge the gap between the equilibrium and ultrafast non-equilibrium properties of MPTS in solution, providing a more complete understanding of photoexcitation energy dissipation pathways through a H-bonding network with a relatively rigid and intact solute. The elucidation of excited-state relaxation and fluorescence mechanisms will help future molecular engineering efforts in site-specific modifications to achieve targeted photophysical and photochemical functions of chromophores and other light-sensitive molecular systems.

## SUPPLEMENTARY MATERIAL

See the supplementary material for the experimental materials, steady-state and ultrafast spectroscopic methods, quantum calculation methods, HOMO and LUMO electron density maps of MPTS in water, global analysis of the fs-TA spectra in three solvents, control experimental fs-TA data of MPTS in water- or methanol–glycerol binary solvent mixtures, FQY measurements of MPTS in three solvents, the SE band redshift dynamics, and additional analysis of the excited-state Raman mode intensity and frequency dynamics from the time-resolved FSRS experiments of MPTS in water and methanol after 400 nm excitation.
